# Combating the infodemic: COVID-19 induced fake news recognition in social media networks

**DOI:** 10.1007/s40747-022-00672-2

**Published:** 2022-02-18

**Authors:** Shankar Biradar, Sunil Saumya, Arun Chauhan

**Affiliations:** 1 Department of Computer Science and Engineering, Indian Institute of Information Technology Dharwad, Dharwad, Karnataka India; 2 Department of Computer Science and Engineering, Graphic Era University Dehradun, Dehradun, India

**Keywords:** Social networks, Fake news, COVID-19, Machine learning, Voting classifier, Contextual embedding

## Abstract

COVID-19 has caused havoc globally due to its transmission pace among the inhabitants and prolific rise in the number of people contracting the disease worldwide. As a result, the number of people seeking information about the epidemic via Internet media has increased. The impact of the hysteria that has prevailed makes people believe and share everything related to illness without questioning its truthfulness. As a result, it has amplified the misinformation spread on social media networks about the disease. Today, there is an immediate need to restrict disseminating false news, even more than ever before. This paper presents an early fusion-based method for combining key features extracted from context-based embeddings such as BERT, XLNet, and ELMo to enhance context and semantic information collection from social media posts and achieve higher accuracy for false news identification. From the observation, we found that the proposed early fusion-based method outperforms models that work on single embeddings. We also conducted detailed studies using several machine learning and deep learning models to classify misinformation on social media platforms relevant to COVID-19. To facilitate our work, we have utilized the dataset of *“CONSTRAINT shared task 2021”*. Our research has shown that language and ensemble models are well adapted to this role, with a 97% accuracy.

## Introduction

Because of digitization, the Internet has become an integral part of our daily lives in recent years. The latest survey reveals that more than 3.8 million people use social media; this is more than half of the world population^1^. These social media networks have given us several benefits, such as enable individuals to produce and share their content, quick and easy connectivity, brand ads, customer reviews, etc. However, it still has many drawbacks, one of which being fake news. Fake (or false) news is defined as news reports that are intentionally, verifiably inaccurate and have the potential to deceive readers [1]. The impact of the false news on society is definitive, direct proof of this being the U.S. election in 2016. During the critical months of the U.S. presidential election campaign, a total of 8,711,000 Facebook posts were discovered from the top 20 often argued fake election reports, which was more than 7,367,000 posts from the top 20 most debated elections shared on 19 major news websites [41]. Fake news also involves violent events in the real world, which jeopardize community peace [15]. Conspiracy theories mainly develop during crises in public health, and cause tremendous effects on life. During the 2019 Ebola outbreak in the Democratic Republic of the Congo (DR Congo), misinformation was led to violence, suspicion, social upheavals, and targeted attacks on healthcare providers [9]. During the 2002–2003 SARS epidemic, uncertainty and anxiety about the disease triggered social stigma toward Asians. Even during the current COVID-19 outbreak, research in the American Journal of Tropical Medicine and Hygiene found that approximately 5,800 patients have been hospitalized due to inaccurate social media information. In addition, many people died of drinking methanol or cleaning drugs dependent on alcohol. They were misinformed that these products are the treatment for the virus [14].

There is a sense of urgency within the scientific community to address fake news on social media. Traditionally, fake news was identified by experts such as journalists^2^. Their primary job is to review allegations against facts based on publicly discussed or published evidence. However, it is time-consuming and costly; furthermore, fake news spreads rapidly, and traditional approaches of dealing with it are ineffective. This is why, the artificial intelligence research community is interested in automating the identification of fake news on the Internet. The aim of automatic fake news identification is to save humans’ time and effort. However, it faces numerous challenges, which we have summarized below.

1. Data from social media are abundant and growing, but its non-structural, incomplete, and noisy nature makes processing and understanding extremely difficult [5].
2. Because fake news items are meant to deceive readers rather than provide objective claims, state-of-the-art methods for identifying false content fall short of better accuracy [5].
3. Fake news may cover a variety of issues such as politics, finance, religion, and health. It can also use various linguistic styles; thus, features that distinguish one type of fake news may not work well for another.

With the automatic false news identification problem in mind, significant efforts have been undertaken to address these difficulties from various viewpoints. Some of these tactics are discussed in the next section.

Our research on social media material is primarily limited to the subject of COVID-19, since the pandemic created an immediate need to build resources to protect against disinformation dissemination. This paper performs detailed studies utilizing machine learning models, such as traditional machine learning models, neural network models, and specific state-of-the-art transfer learning models for false information classification in the COVID-19 data set.

The main contribution of the paper are:

1. To develop an early fusion-based approach for combining key features extracted from context-based embeddings to improve the efficacy of fake news identification.
2. To develop various Recurrent Neural Network models, such as LSTM, BiLSTM, GRU, and BiGRU, with pre-trained BERT embeddings for fake news identification. In addition, their ensemble settings are also explored.
3. To develop a hybrid voting classifier by integrating the results of conventional machine learning algorithms and language models.
4. To develop a multi-level bit-wise operator-based classifier for fake news identification.

The rest of the article is arranged as follows. The specifics of past work done are given in Sect. 2. In Sect. 3, the implementation specifics are outlined. We presented our results and discussions in detail in Sects. 4 and 5. Subsequently, Sect. 6 offers the conclusion and scope for the future.

## Related works

The ever-increasing attraction and beauty of using social networks directly or indirectly influence our everyday lives. Therefore, it is not shocking that social networking has been a platform to exploit emotions through disseminating misinformation according to patterns. The adverse use of these channels was primarily used to spread inaccurate or unclear, communal, financial, and political information^3^. For example, false news research on Twitter in a Boston attack reveals that scare peddlers successfully manipulate social media to cause mass hysteria and panic [11]. Furthermore, distributing disinformation may have a detrimental effect on individuals and the communities they work in; it can instill fear and affect their emotional reactions to elections and natural disasters [3, 18]. The spread of misleading vaccine information has been one such example, indicating that many parents have spread fake news concerning the vaccine’s safety. Consequently, some young children’s parents have criticized the pediatric advice and declined to vaccinate or immunize their children [20]. As a result, there has been an alarming increase in sickness that could have been avoided.

In recent years, the research community has been exploring the driving force behind the spread of false information on incidents such as the pandemic COVID-19 or policy scenarios [26, 36] has uncovered early social media campaigns on political, religious, and economic propaganda. Results revealed that hacked identities (using someone’s personal information and pictures to create fake profiles and use them to spread fake information [30, 31]) are used to promote misinformation and can also be used to propagate propaganda [7] attempted to track the dissemination of scientific misinformation, which concluded that most people found details reliable, because their friends also tweeted. Another potential cause is that people want to share new information [38].

### Fake content detection in various scenarios

Avoiding the spread of fake news has been a serious concern for the scientific community. Recently, a significant amount of research has been conducted to identify fake information on social media. Fake news identification methods are typically classified into four classes: (1) news content-based; (2) social background-based [34, 43]; (3) propagation-based; and (4) information-based.

Social background-based methods incorporate elements from social media user accounts and message content [32] attempted to construct interaction networks that represent the interconnections between various entities such as publishers (person who post the news), news articles, and users to perform fake news detection [6] proven that attributes gathered from user posting and re-posting behaviors will help in determining the reliability of the information [35] tried to combine post-content with social context information such as relationships among the publishers and users.

Propagation-based models study the fake news spreading pattern in Social Media Networks. [19] have extracted information from social networks (structural properties of fake news spreading) to identify fake news; they built a friendship network for this purpose. [28] built an RNN-based framework consisting of three models: the first model captures the temporal patterns of user activity on a given post. The second module learns the source characteristic from user behavior, and the third module integrates the two [33] have developed hierarchical dissemination networks for false news and real news and then conducted a comparative review of structural, temporal, and linguistic networking features for the perspective on recognizing fake news. The majority of these models can be grouped as positions or propagation-based. However, the issue with these approaches is the early identification and prevention of the dissemination of fake news.

Some analysis experiments have been conducted to establish the basis for false news studies with information base and knowledge diagrams, such as DBpedia and Knowledge Vault [46]. They depend on an established information base that includes “common knowledge” to equate news articles with the truthfulness of the news. However, recently emerging methods often produce new and unforeseen information, which is not contained in the current knowledge bases or knowledge diagrams. Therefore, such approaches are often incapable of dealing with news stories [46]. One more alternative is to use a credibility-based approach; these methods require external content, such as source information and news comments, to determine false news, which may not always be available, especially if the item is spread via social media [46].

Content-based approaches extract various features from news content and are better adapted for early fake news detection. [21] used a content-based machine learning approach; the author employed two types of machine learning models. The first is the word-bag model, made more robust by stacking two layers of ensemble learning models. Second, neural networks that use pre-trained GloVe Word embeddings, including (a) a one-dimensional convolutional neural network (CNN) and (b) a bi-directional long short-term memory network (BiLSTM); [27] tried to detect fake news by constructing deep semi-supervised two-way CNN. Machine-generated fake news detection was explored by [45]. More recently, [40, 44] have tried to combine user comment emotion with post-content to improve the accuracy of prediction. Finally, [17] proposed a multi-layer dense neural network for identifying fake news in Urdu news articles.

### Fake content detection on COVID-19 data

In the previous research, the primary subject was political and communal fake news spread through social media. However, very few studies have concentrated on spread of disinformation, linked mainly to COVID-19. [10] tried to combine topical Dirichlet Allocation (LDA) with contextualized representations from XLNet for the task of fake news identification related to COVID-19. [8] achieved 95% accuracy by applying standard machine learning algorithms on many linguistic features such as n-grams, readability, emotional tone, and punctuation for the COVID data set. [24] presented CTF, a large-scale COVID-19 Twitter dataset with tagged real and fake tweets. Cross-SEAN, a semi-supervised end-to-end neural attention model based on cross-stitching, was also proposed. They obtained an F1 score of 0.95% percent on the CTF data set. The usefulness and effectiveness of pre-trained Transformer-based language models in retrieving and classifying fake news in a specialized domain of COVID-19 were demonstrated by Vijjali et al. [37]. They also concluded that the suggested two-stage model outperforms other baseline NLP techniques. Gupta et al. [12] developed a model to detect fake news about COVID-19 in English tweets. They attain a 0.93 F1 score on the English fake news detection challenge by combining entity information taken from tweets with textual representations learned through word embeddings. Finding fake news related to COVID-19, on the other hand, is a “need of the hour” during this pandemic, and a lot of research needs to be conducted on this topic.

## Methodology

This section explains the different learning models developed and experimented for COVID-19 fake news identification. We first discuss the data set used in the study, and later, its pre-treatment and different fake news classifiers are explained in detail.

### Dataset description

We have used the dataset released in the “*CONSTRAINT shared task-2021*” for the current study [25]. The dataset included real news as well as fake news regarding the COVID-19 issue in social media. The total number of real news samples are 5600, and fake news samples are 5100. The data set has two fields: tweet and label, and it is balanced overall. Fake news were gathered from numerous web pages, e.g., Politifact1, NewsChecker2, Boomlive3, etc., using tools such as IFCN chatbot5 and Google Reality Search Explore4. In addition, the real news were gathered from Twitter using verified Twitter handles [25]. The detailed distribution of the dataset is shown in Table 1, while sample sentences from the dataset are shown in Table 2.

**Table 1:** Dataset distribution

Split	Real	Fake	Total
Train	3360	3060	6420
Validation	1120	1020	2140
Test	1120	1020	2140
Total	5600	5100	10700

**Table 2:** Sample sentences from dataset

Sentence	Label
The CDC currently reports 99031 deaths.	Real
In general, the discrepancies in death counts between different	
sources are small and explicable. The death toll stands at roughly	
100000 people today.	
CDC Recommends Mothers Stop Breastfeeding To	Fake
Boost Vaccine Efficacy	
The WHO confirmed asymptomatic persons can’t	Fake
transmit the coronavirus and are not infectious	
The confirmation earlier today of a second death	Real
linked to COVID-19 in the last two days means the number of	
COVID-19-related deaths in New Zealand are now 24.	

### Data pre-treatment

A few data pre-treatment steps were taken on both text and label fields to process the data for model training. The textual corpus included URLs, hyperlinks, figures, stop words, and capital letters. Various pre-processing exercises were performed to simplify details, such as replacing punctuation with white spaces, removing the URLs, and Twitter user handles that did not help to identify fake news. Converting texts into the lower case was also done to remove the redundant tokens. Furthermore, the data lemmatization was carried out to translate the term into its useful basic form, such as the words runs, running, and ran are all mapped to their root word run. The tokenizer was then fed with pre-processed text to convert each tweet into the number of tokens where each word in the tweet was assumed to be a single token. In combination with tokenization, padding and masking for the variable-length phrases were also performed. The padding ensures that all the input sequence data have the same length by padding or truncating the input data points. The masking removes the importance of padded tokens during model construction by setting the padded value to zero. Our model used Bi-directional Encoder Representations (BERT) and generalized autoregressive pre-training method (XLNet) tokenizers.

### Embedding

We used various contextual embedding techniques such as DistilBERT, a generalized autoregressive pre-training method (XLNet), and Embeddings from Language Models (ELMo) to preserve the sentence context. Three different embedding were used, because we wanted to test the performance of our proposed models on different context-based embeddings. The DistilBERT is pre-trained as a smaller general-purpose language model, reducing the complexity of the BERT model by 40% while maintaining 97% of their language comprehension and 60% faster [29]. DistillBERT’s design is similar to the original BERT and comprises 12 self-attention heads and 12 transformer blocks with a hidden layer size of 768. We only drew the special [CLS] token present at the start of the DistilBERT model for classification, which provides full-sentence embedding. The XLNet is another model based on a transformer that uses autoregression and autoencoding techniques. Like BERT, it has 12 layers, 12 attention heads with 768 hidden layers, and is trained on 110 million parameters. However, the [CLS] token is present at last in this model, which gives the full-sentence embedding [42]. ELMo is one more state-of-the-art pre-trained contextual-based embedding technique that uses a deep, bi-directional LSTM model to create word representations. It generates embeddings of dimension 1024 [23]. The embedding representations used by ELMo are characteristics of the complete input sentence. They are computed as a linear function of the internal network states on top of two-layer BiLSTM with character convolutions. ELMo employs the semi-supervised learning principle. The most fundamental advantage of using pre-trained embeddings like BERT, XLNet, and ELMo in our proposed model is that they are trained on a massive corpus and works well for smaller training data set. Therefore, we tried to evaluate the performance of different machine learning and deep learning models using pre-trained embeddings.

### Classification

The main objective of this work is to construct a classifier that separates our corpus into a real or fake class. Several machine learning models, neural network models, and a few language models were used for this purpose. We have broadly categorized our proposed models into four different categories: (1) Early fusion-based DNN model, (2) RNN-based ensemble model, (3) Voting classifier model, and (4) Multi-level bit-wise operator model. The reason behind using the aforementioned model in our proposed approach is that in our experiments, these models performed better than the other models, such as traditional machine learning and simple feed-forward neural network models, and the results of those are shown in Table 4.

**Fig. 1:**
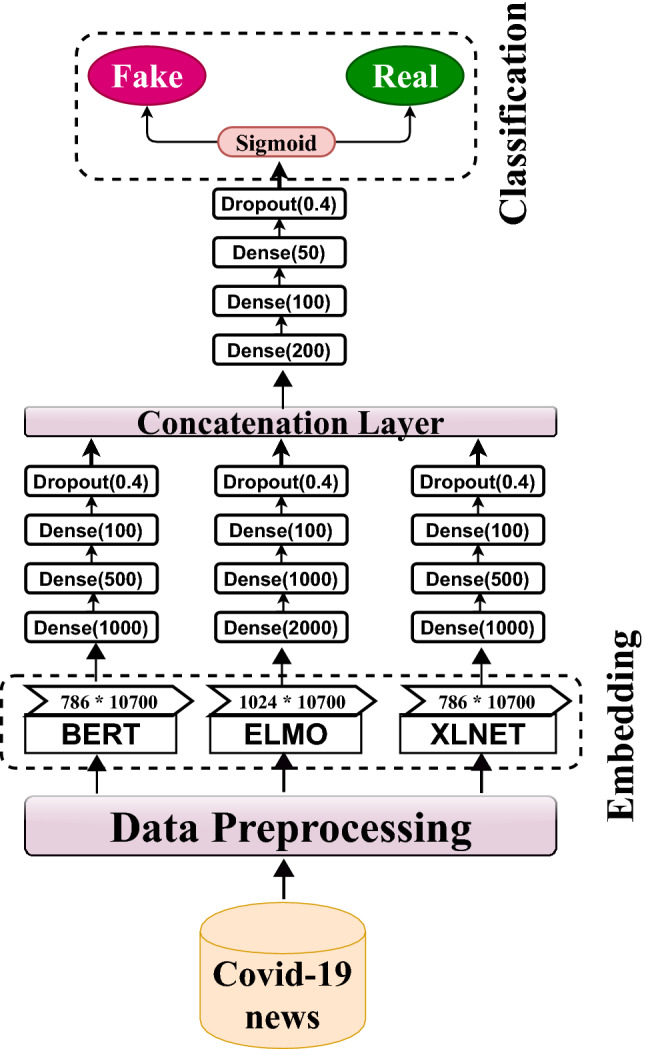
Early fusion-based DNN model architecture


In **Model 1**, we used an early fusion-based approach to combine key features from input sentence embeddings. This model used three parallel sub-models to extract features from embeddings such as BERT, XLNet, and ELMo. Initially, input from the embedding layer is passed through Dense layers of sizes 1000, 500, and 100 for BERT, XLNet, and 2000, 1000, and 100 for ELMo to extract key features from the embedding vector. Then, batch normalization and a dropout rate of 0.4 are added to prevent overfitting issues. The grid search is used to find the optimal hyperparameter value of dropout that results in the most accurate prediction. We performed a grid search with a dropout rate range of 0.1–0.8 and discovered that a dropout rate of 0.4 produces better results in our suggested model. Next, the output of these dropout layers is concatenated using the concatenation layer. The concatenated output is then passed through several dense layers with dropout before being used for classification in the sigmoid layer. The detailed architecture of Model 1 is presented in Fig. 1, and the pseudocode for the same is given in Algorithm 1. All these models were built using Python’s sci-kit-learn^4^ and transformer library^5^.

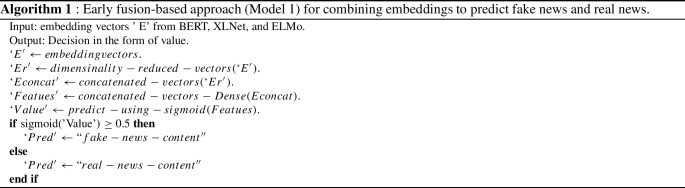


**Fig. 2:**
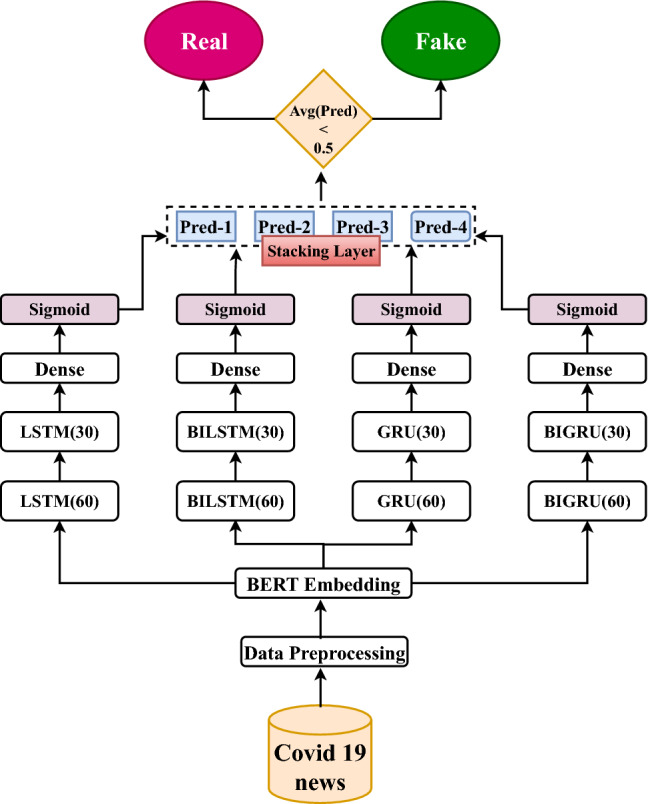
RNNs’ ensemble model architecture


In **Model 2**, multiple RNN variants such as LSTM, BiLSTM, GRU, and BiGRU with two stacked layers of 60 and 30 neurons were developed on the features extracted from BERT embeddings. The LSTM model is used in the proposed approach to resolve the vanishing gradient problem of RNN models. We also experimented with bi-directional models such as BiLSTM and BiGRU in our suggested approach to explore information in both directions, as the prediction of the current word is sometimes dependent on the presence of next and previous words. Furthermore, GRU was chosen, because it has a lower computational cost than LSTM. We used the Adam optimizer with a loss function of ’binary-cross entropy’ and a dropout value of 0.2 in our experiment. Constant values of 60 and 30 neurons are selected from experimental trials, whereas dropout 0.2 is determined using a grid search on a dropout rate range of 0.1–0.8. In our experiment, we used the *Adam* optimizer, because it converges faster than other optimizers and performs better on noisy social media data. We also experimented with the ensembled setting of base learner models, wherein the probability values of all the base learner’s predictions are stacked into a list. The probability values of base learners’ predictions were then added up and divided by the number of base Learners to get the average prediction value. The final prediction is obtained by comparing the average prediction to a threshold value of 0.5. Figure 2 illustrates the Model 2 architecture in detail. The model was developed using Python’s sci-kit-learn library.

**Fig. 3:**
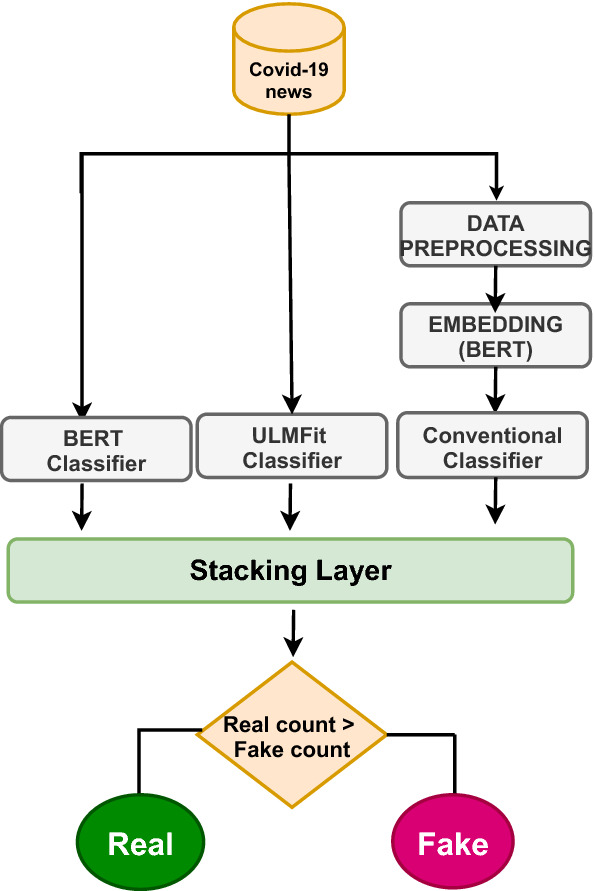
Voting classifier architecture

In **Model 3**, We attempted to combine the results of traditional machine learning algorithms with state-of-the-art language model classifiers such as BERT and ULMFit by employing the voting classifier. To build BERT and ULMFit model, we have used simple classification API provided by the developers. Bert-base-uncased and ASGD Weight-Dropped LSTM (AWD-LSTM) is our underlying option model for BERT and ULMFit, respectively. AWD-LSTM is a state-of-the-art language model composed of regular LSTM with no attention. AWD-LSTM consists of three stages: LM pre-training in which a language model is trained with a large wikitext-103 corpus to capture general features of language. LM fine-tuning in which the model is fine-tuned to the target task data. Finally, the classifier is fine-tuned using the BPT3C language model [13]. In our proposed model initially, the base learners’ binary prediction values are stacked into a single list, and the final outcome is selected based on the majority prediction value. In this approach, we employed “hard voting” to divide the corpus into Real and Fake categories. For example, if predictions from base learners are [1 1 0], then the final prediction based on majority count will be 1. The architecture of model 3 is depicted in Fig. 3, and pseudocode for the voting classifier model is given in Algorithm 2. The huggingface^6^ library in Python is used to build the BERT model, while the fastai^7^ library is used to create the ULMFit model.

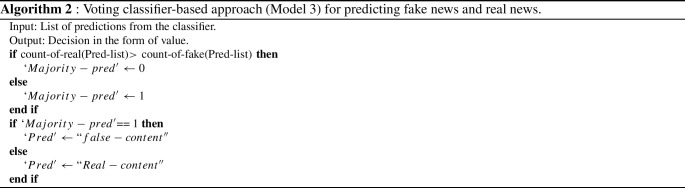

**Fig. 4:**
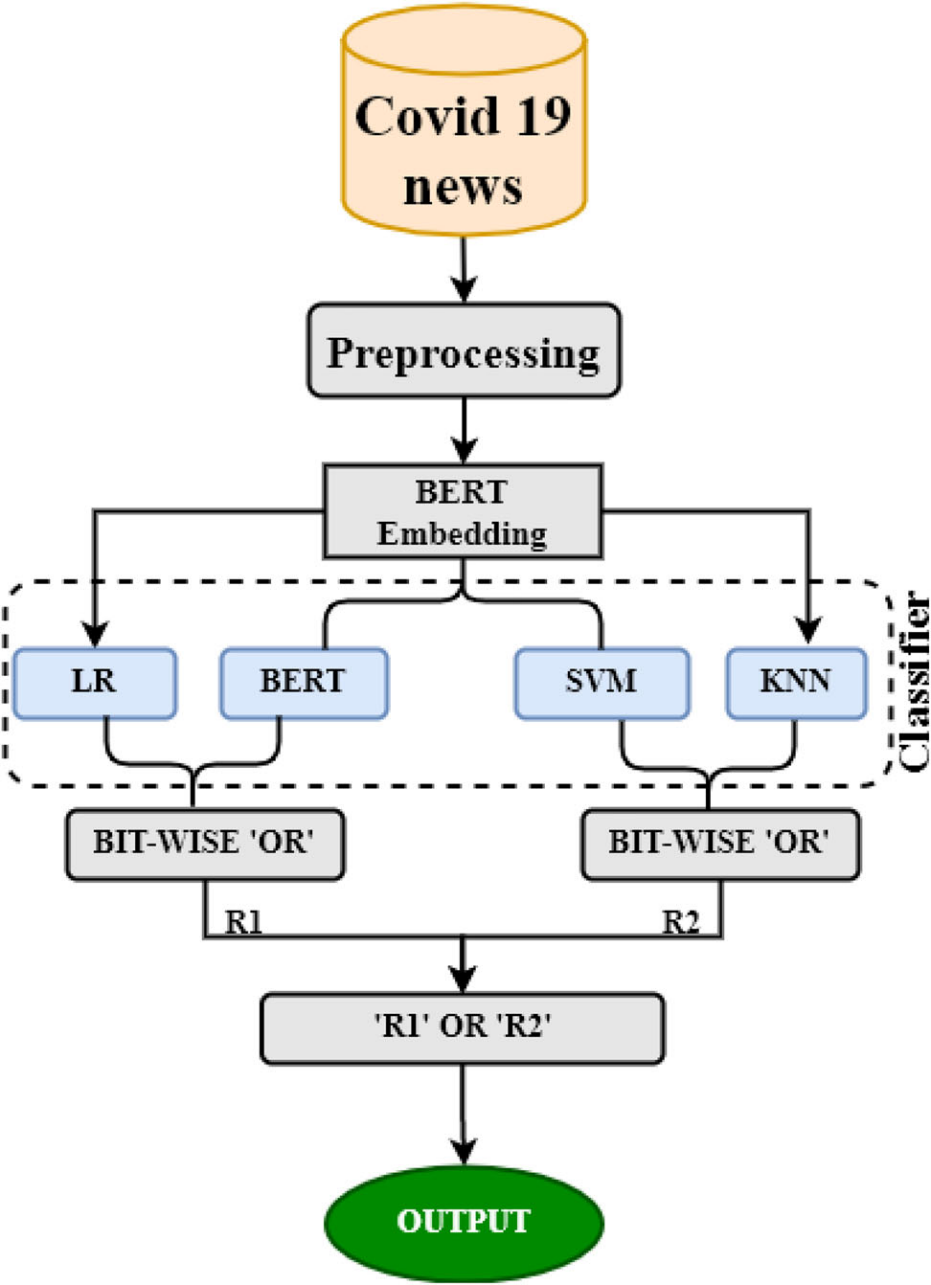
Multi-level bit-wise OR model architecture


Finally, in **Model 4**, we experimented with a bit-wise ’OR’ operator to combine the results of traditional ML models and language models. We used a multi-level ’OR’ing operation in this approach. Initial findings from base learners LR, BERT, SVM, and KNN are binary predictions like as 1s and 0s, which are then merged using the ’OR’ operator to provide intermediate results ’R1’ and ’R2’. These intermediate outcomes are later combined to obtain the final prediction using one more ’OR’ operator. For example, if base learner predictions are (1 0 0 1), the final prediction after passing through the ’OR’ operator is 1, wherein 1 represents fake and 0 means real (Fig. 4). The reason for adopting the aforementioned models as base learners is because BERT is the highest performing model among language models, and LR, SVM, and KNN perform better among traditional machine learning models, as illustrated in Tables 4 and 7.

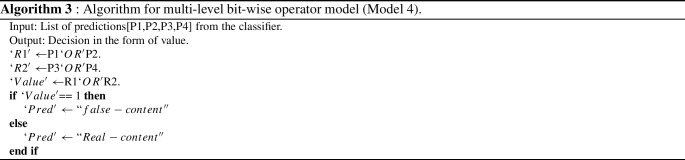


### Optimizers and loss functions

We used *Adam* optimizer in our all proposed neural network models as it incorporates the sparse gradient benefits of AdaGrad with the ability of RMSProp to solve non-stationary objectives. In addition, *Adam *is stable and well suited to various problems [16]. Since the current study deals with a binary classification problem, we used binary cross-entropy as a loss function.

**Table 3:** Parameters for ML models

Classifier	Hyperparameter
Logistic regression	C=1, max-iter=500
Random forest	no-of-estimators=200
Naive bayes	var-smoothing=1e-09
Support vector machine	c=1, solver=‘lbfgs’
K nearest neighbors	n-neighbors=24

**Fig. 5:**
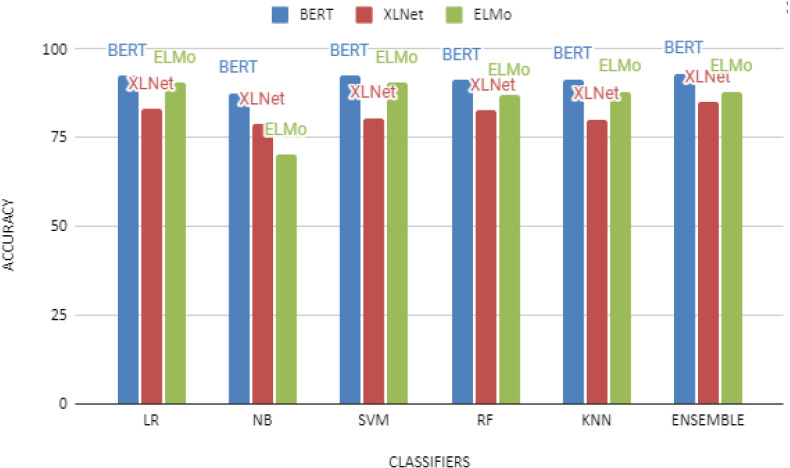
Comparative analysis of ML algorithms on different embedding

**Table 4:** Test set results of traditional algorithms on different embedding

	BERT Embedding		XLNet Embedding		ELMo Embedding	
Classifier	Acc	*F*1-score	Acc	*F*1-score	Acc	*F*1-score
		0.92-Fake		0.82-Fake		0.90-Fake
LR	0.9257		0.8313		0.9038	
		0.93-Real		0.84-Real		0.90-Real
		0.87-Fake		0.78-Fake		0.71-Fake
NB	0.8759		0.7886		0.70166	
		0.88-Real		0.79-Real		0.69-Real
		0.92-Fake		0.79-Fake		0.90-Fake
SVM	0.9241		0.8053		0.9044	
		0.93-Real		0.83-Real		0.90-Real
		0.91-Fake		0.82-Fake		0.87-Fake
RF	0.9123		0.8273		0.8694	
		0.91-Real		0.84-Real		0.86-Real
		0.91-Fake		0.79-Fake		0.86-Fake
KNN	0.9143		0.8012		0.88	
		0.91-Real		0.81-Real		0.87-Real
		0.93-Fake		0.84-Fake		0.87-Fake
ENSEMBLE	0.93		0.85		0.88	
		0.93-Real		0.85-Real		0.88-Real

## Results

Let **‘S’** be the set of social media posts. For each social media post **‘P’**, ${(\forall P\in S)}$, we have two independent sets: training set** {Sx,Sy}** and testing set **{Sp,Sq}**. We aim to apply information gained from the training set** {Sx,Sy}** to conduct effective fake news detection on the corresponding test set **{Sp,Sq}**. We tested our models with two matrices: Accuracy and F1 score. Accuracy can provide a measure of all accurate classifications, but we also interested to know the measure of incorrect classifications to analyze the performance of our model on both fake and non-fake data; hence, we used both F1 score and accuracy as evaluation matrices. The mathematical expression for aforementioned matrices are as follows:

*Precision (P) = W/W+X*

*Recall (R) = W/W+Y*

*Accuracy = W+Z/N, where N=W+X+Y+Z * > 0

F-1 Score = 2*P*R/P+R,

where **‘W’** represents the true-positive score **(TP)**, **‘X’** represents false-positive score **(FP)**, **‘Y’** represents false-negative score **(FN)**, and **‘Z’** represents true-negative score **(TN)** respectively.

### Initial experimental results

Initial experiments were performed using conventional machine learning algorithms, such as Logistic Regression, Random Forest, Naive Bayes, Support Vector Machine, and K nearest neighbors (tested with 24 different values for n-neighbors and then developed our grid search according to that value). Table 3 presents the parameter used during the training for the aforementioned classifiers. These classifiers have been investigated using BERT, XLNet, and ELMo embeddings. The results obtained from these models are illustrated, as shown in Fig. 5. These results are also summarized in Table 4. It can be observed from Table 4 that LR achieves the highest accuracy of 92.57% and 83.13% for BERT, and XLNet embeddings and SVM outperformed other models with 90.44% accuracy for ELMO embeddings. Finally, we used ensemble learning to combine predictions from all classifiers for all three embeddings, and the results are shown in the last row of Table 4. As can be seen, the overall accuracy is improved to 93% for BERT, 85% for XLNet, and 88% for ELMo embeddings.

**Fig. 6:**
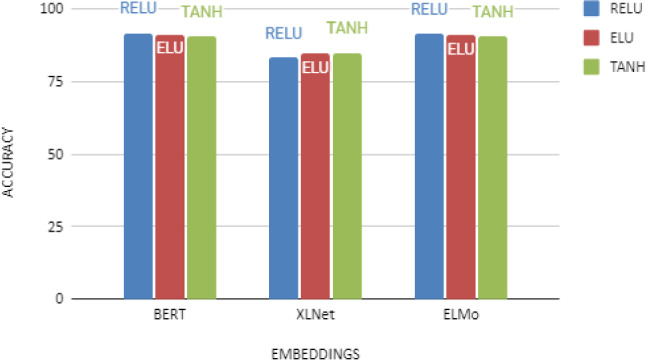
Performance analyses using different activation function

Next, the attempt was made to examine the efficiency of a basic feed-forward neural network on a separate embedding with two hidden dense layers, L1=60 and L2=50 neurons. We further explored the influence of the various activation functions on the outcomes. The findings are indicated in Fig. 6. It is observed from Fig. 6 that RELU activation function performs better than the remaining for all three embeddings.

### Proposed model results

In **Model 1**, we assessed our model’s performance on various context-based embeddings, such as BERT, XLNet, and ELMo. We later incorporated key features from these three embeddings into a single vector by employing several dense layers with a concatenation layer and classified the resulting vector. Table 5 represents the outcomes of Model 1 on the test data set. From the table, classifiers such as BERT embedding+DNN, ELMo embedding+DNN, and XLNet embedding+DNN achieved an accuracy of 91.58%, 92.16%, and 83.13%, respectively. The combined model of BERT+XLNet+ELMo embeddings + DNN achieved an accuracy of 93%. The outcome in Table 5 clearly shows that integrating the key features from all three embeddings will increase the classifier’s efficiency on our test data set.

In **Model 2**, the efficiency of RNN-based models for the classification of the COVID-19 dataset was explored. Initially, we performed individual experiments with different variants of RNN. Later, to increase model performance, we experimented with their ensemble setup, which calculated the average prediction value using the probability values of base learner predictions, as described in “Classification”. The final result is determined by comparing the average prediction to the threshold value. The results tabulated in Table 6 showed that the ensemble model outperformed the remaining models with an accuracy of 92%, and the next best performing model is BIGRU with an accuracy of 91.978%.

**Table 5:** Test set results of early fusion-based DNN models **(Model 1)**

Classifier	Acc (%)	*F*1-score (%)
BERT embedding+DNN	91.58	92-Fake, 91-Real
ELMO embedding+DNN	92.16	92-Fake, 92-Real
XLNet embedding+DNN	83.13	83-Fake, 84-Real
BERT+ELMo embeddings+DNN	92.61	93-Fake, 92-Real
XLNet+BERT embeddings+DNN	90.66	90-Fake, 89-Real
XLNet+ELMo embeddings+DNN	91.93	91-Fake, 92-Real
BERT+XLNet+ELMo embeddings+DNN	93	93-Fake, 92-Real

**Table 6:** Test set results of RNN-based models **(Model 2)**

Classifier	Acc (%)	*F*1-score (%)
LSTM	91.74	91-Fake,92-Real
BILSTM	91.121	91-Fake,91-Real
GRU	90.23	89-Fake,91-Real
BIGRU	91.978	91-Fake,93-Real
Ensemble	92	92-Fake,93-Real

In **Model 3**, for our task of classifying the COVID-19 data collection, first, we worked with language model classifiers such as BERT and ULMFit. The BERT and ULMFit developers provide a simple classification API. Bert-base-uncased and ASGD Weight-Dropped LSTM (AWD-LSTM) [22] is our underlying option model for BERT and ULMFit, respectively. From the initial findings, we found that the BERT classifier outperforms the ULMFit classifier with an accuracy value of 97%, as shown in Table 7. Next, we attempted to combine the outcomes of a language model with output from conventional machine learning algorithms using the voting classifier model, as illustrated in Fig. 3. From Table 7, it can be seen that the ensemble setting of LR, ULMFit classifier, and BERT classifier has achieved the highest accuracy of 98%.

**Table 7:** Test set result of language and voting classifier model models **(Model 3)**

Classifier	Acc (%)	*F*1-score (%)
BERT classifier	97	97-Fake,98-Real
ULMFit classifier	96	96-Fake,96-Real
LR,ULMFit classifier, BERT classifier	98	98-Fake,98-Real
LR,KNN,BERT classifier	96	96-Fake,96-Real
LR,SVM,RF,KNN AND BERT classifier	95	95-Fake,95-Real

**Table 8:** Test set result for bit-wise operator models **(Model 4)**

Model	Acc(%)	*F*1-score(%)
LR OR BERT	95	94-Fake, 95-Real
ULMFit OR BERT	96	96-Fake, 96-Real
( LR OR BERT) OR (SVM OR KNN)	92	92-Fake,93-Real

In **Model 4**, to further improve the efficiency of our proposed models, we examined the effect of the bit-wise ‘OR’ operator on the outcomes. The results are tabulated in Table 8. As shown in Table 8, bit-wise ’OR’ing between ULMFit and BERT classifiers achieved the maximum accuracy of 96%. We also tried multi-level ‘OR’ing between the outcomes of traditional machine learning algorithm with those of the BERT classifier, and the model achieved an accuracy of 92% as shown in the last row of Table 8.

### Additional experiments

**Fig. 7:**
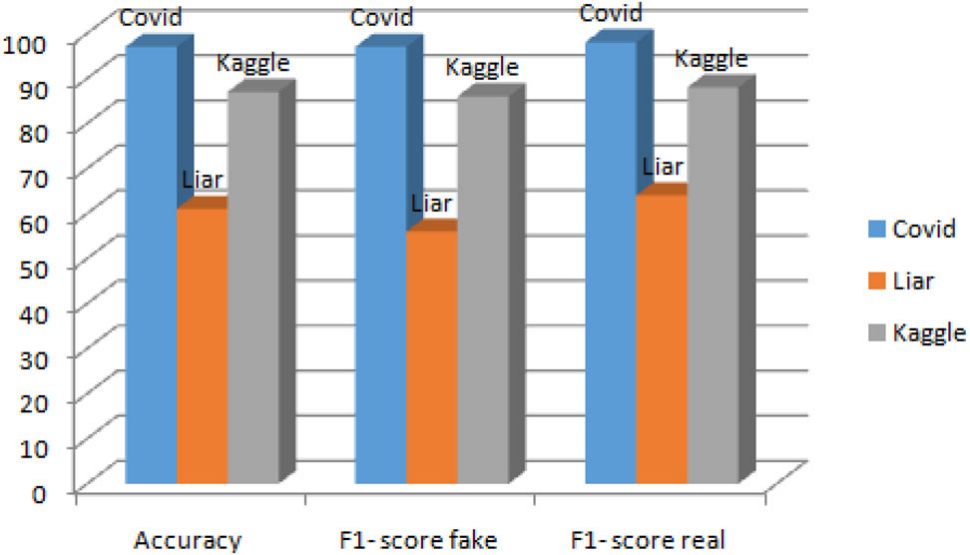
Performance analyses using different data set

To assess the performance of the proposed model, we conducted experiments on two additional social media data sets, such as liar [39] and Kaggle fake news data set^8^, using our best-performing model, and the results are summarized in Fig. 7. The experimental results show that the proposed model performs better on both data sets, with an accuracy value of 87% on the Kaggle dataset and 61% on the Liar dataset, allowing it to be used as a general solution for identifying fake news in any domain.

We also investigated the behavior of our proposed models on sample sentences from the test data set and discovered that the majority of proposed models were able to classify it correctly. Table 9 displays the results of the test cases. As shown in Table 9, all proposed models accurately classified the first test sample. Still, for test samples 2 and 3, Model 1 and Model 2 could not predict the actual target value. However, Models 3 and 4 accurately classified the target value in all three cases, owing to their superior performance. Some sample sentences containing emotions (test samples 4 and 5) were also included in the study, and it was discovered that the majority of the models correctly classified those sentences. Finally, to take advantage of domain-specific embeddings, we tested traditional machine learning classifiers with embeddings from ClinicalBERT [2], which was trained on electronic health records of ICU patients, and the results are summarized in Table 10. The architecture of ClinicalBERT is similar to that of the original BERT, as mentioned in Sect. 3. However, due to a lack of medical terminology in our training data set, domain-specific embeddings failed to outperform conventional BERT in our initial trials. Therefore, we do not proceed with the proposed models.

**Table 9:** Test cases for fake news

Sample text	Model-1	Model-2	Model-3	Model-4	Target
Bill Gates said that the COVID-19 vaccine will permanently change your DNA	Fake	Fake	Fake	Fake	Fake
COVID-19 is caused by a bacterium, not virus and can be treated with aspirin	Fake	Real	Fake	Fake	Fake
EMA endorses the use of dexamethasone for COVID-19	Fake	Fake	Real	Real	Fake
Thank God! new COVID-19 clusters mostly affecting low paid workers	Fake	Fake	Fake	Fake	Fake
A video of a television presenter where she says “thank Godwhere she says “thank God things get complicated” referring to the coronavirus in Germany to the coronavirus in Germany	Real	Fake	Fake	Fake	Fake

**Table 10:** Results on domain-specific embeddings

	Embedding	Accuracy(%)	*F*1-score fake(%)	*F*1-score real(%)
LR	ClinicalBERT	88	88	89
NB	ClinicalBERT	80	80	80
SVM	ClinicalBERT	90	90	90
RF	ClinicalBERT	85	85	86
KNN	ClinicalBERT	85	85	86
Ensemble	ClinicalBERT	86	85	86

**Table 11:** Comparative analysis our model with some existing models

Source	Model	Acc (%)	*F*1-score (%)
[8]	SVM+linguistic features	95.19%	95.70
[4]	ladiff ULMFit	96.72	96.73
[10]	XLNet with topic distributions	96.8	96.7
proposed model1	early fusion-based model	93	93
proposed model2	RNN-based model	92	92
**proposed model3**	**LR,ULMFit,BERT Classifier**	**97**	**98**
proposed model4	bit-wise operator-based model	96	96

## Discussion

From the results in the previous section, we have found that the logistic regression (LR) and Support Vector Machine (SVM) are the best-performing models among the conventional machine learning algorithms. Among the word embeddings, BERT performed better than XLNet and ELMo from Table 4, indicating that BERT handles our tweeter data better. The performance of machine learning models utilizing XLNet word embedding was unexpected. The performance of XLNet was poor, which could be attributed to the fact that our dataset contained short sentences, whereas XLNet is trained to handle long sentences. In addition, our early fusion-based model for combining the key features from three different embedding vectors(Model 1) enhanced the classifier’s efficiency on our test data set, as shown in Table 5. In Model 2, among the different variants of RNN models, BiGRU performed better than other models. However, the best performance was reported by their Ensemble setting with 92% accuracy (shown in Table 6).

Our benchmark findings on various machine learning models show that the Voting Classifier model comprised LR, BERT, and ULMFit were the best-performing models for detecting false news in our COVID-19 dataset (Model 3). They achieved an accuracy of 98% with the F-1 score of 98% for fake class and 98% for real class. BERT and ULMFit Classifiers followed it with an accuracy of 97% and 96%, respectively, as shown in Table 7. As a result, the overall study concluded that state-of-the-art language models and ensemble models outperform other machine learning techniques in recognizing fake news in the COVID-19 dataset. Our findings show that language models like BERT and ULMFit fared better on our data set, because they were trained on massive Wikipedia corpora. We also compared our work with some existing models on the same data set, and the result is indicated in Table 11. As shown in Table 11, [8] have used SVM classifier with linguistic features extracted from a tweet and reported an accuracy of 95%. [4] used a Layer Differentiated training approach for ULMFit, and [10] used XLNet embeddings with Topic Distributions to achieve accuracy of 96.7% and 96.8%, respectively. According to Table 11, our model is the best-performing model among the existing models on the same dataset.

### Implications and limitations of our model

Fake news is now a regular phenomenon on social media sites, especially amid health crises; the size of the fake news-related pandemic is increasing at an alarming rate. Therefore, it is critical to stop the spread of fake news. The models provided in this article can help to improve the performance of existing methods for dealing with the propagation of fake news through social media sites. However, even though our models considerably improved the performance of existing methods, none of the models are perfect, and there is always room for improvement. Furthermore, even our model has some limits that can be addressed in future research. Some of our model’s limitations include:

1. Due to computational constraints, our model is trained on a limited data set. Future studies could broaden this to a bigger corpus.
2. Our study did not account for code-mixed and native language statements. Therefore, there is always the possibility of expanding it to include a low resource regional and code-mixed language data set.

## Conclusion and future work

The present COVID-19 pandemic is a threat to people. Much of which will rely on the precision and credibility of shared knowledge to control and prevent COVID-19 among the inhabitants. This article has performed comprehensive research on various machine learning models to classify ‘fake’ news in the COVID-19 dataset and summarized the benchmark findings. Our findings indicated that ensemble and language models are doing better than the other models to classify fake news in our corpus with accuracy value of 98%. Furthermore, among all the embedding discussed, BERT embedding performed better than XLNet and ELMo by a larger margin with the available short text data extracted from Twitter. And also, combining features extracted from different embeddings into a single vector for classification will increase the performance by a small margin. Furthermore, future work can extend our study into multimodal data containing both picture and text pieces. future research can also include mixed language code, and regional languages; this is critical, because many people over the globe rely on the knowledge they exchange in their native language. Additionally, performance of existing methods can be improved by training them on a larger corpus of health-related fake news.

## Footnotes

1 https://www.broadbandsearch.net/.

2 https://www.politifact.com/.

3 http://www3.weforum.org/docs/GRR17_Report_web.pdf.

4 https://scikit-learn.org/stable/.

5 https://huggingface.co/docs/transformers/index.

6 https://huggingface.co/docs/transformers/index.

7 https://docs.fast.ai/tutorial.text.html.

8 https://www.kaggle.com/jruvika/fake-news-detection.

## References

[1] Allcott H, Gentzkow M (2017). Social media and fake news in the 2016 election. J Econ Perspect.

[2] Alsentzer E, Murphy JR, Boag W, Weng W-H, Jin D, Naumann T, McDermott M (2019) Publicly available clinical BERT embeddings. In: Proceedings of the 2nd clinical natural language processing workshop, pp 72–78

[3] Anderson J, Rainie L (2017) The future of truth and misinformation online. Pew Research Center, pp 1–224

[4] Azhan M, Ahmad M (2021) LaDiff ULMFiT: a layer differentiated training approach for ULMFiT. In: Chakraborty T, Shu K, Bernard HR, Liu H, Akhtar MS (eds) Combating online hostile posts in regional languages during emergency situation, vol 1402. Springer, Cham, pp 54-61. 10.1007/978-3-030-73696-5_6

[5] Cao J, Guo J, Li X, Jin Z, Guo H, Li J (2018) Automatic rumor detection on microblogs: a survey. arXiv e-prints arXiv:1807.03505

[6] Castillo C, Mendoza M, Poblete B (2011) Information credibility on twitter. In Proceedings of the 20th International Conference on World Wide Web, pp 675–684

[7] De Domenico M, Lima A, Mougel P, Musolesi M (2013). The anatomy of a scientific rumor. Sci Rep.

[8] Felber T (2021) Constraint 2021: machine learning models for COVID-19 fake news detection shared task. arXiv e-prints arXiv:2101.03717

[9] Fung IC-H, Fu K-W, Chan C-H, Chan BSB, Cheung C-N, Abraham T, Tse ZTH (2016). Social media’s initial reaction to information and misinformation on Ebola, august 2014: facts and rumors. Public Health Rep.

[10] Gautam A, Venktesh V, Masud S (2021) Fake news detection system using XLNet model with topic distributions: CONSTRAINT@AAAI2021 shared task. In: Chakraborty T, Shu K, Bernard HR, Liu H, Akhtar MS (eds) Combating online hostile posts in regional languages during emergency situation, vol 1402. Springer, Cham, pp 189–200. 10.1007/978-3-030-73696-5_18

[11] Gupta A, Lamba H, Kumaraguru P (2013) \$1.00 per rt# bostonmarathon# prayforboston: analyzing fake content on twitter. In 2013 APWG eCrime Researchers Summit, IEEE, pp 1–12

[12] Gupta A, Sukumaran R, John K, Teki S (2021) Hostility detection and Covid-19 fake news detection in social media. arXiv e-prints arXiv:2101.05953

[13] Howard J, Ruder S (2018) Universal language model fine-tuning for text classification. In: Proceedings of the 56th annual meeting of the association for computational linguistics, vol 1: Long Papers, pp 328–339

[14] Islam MS, Sarkar T, Khan SH, Kamal A-HM, Hasan SM, Kabir A, Yeasmin D, Islam MA, Chowdhury KIA, Anwar KS (2020). COVID-19-related infodemic and its impact on public health: a global social media analysis. Am J Trop Med Hyg.

[15] Kang C, Goldman A (2016) In washington pizzeria attack, fake news brought real guns. New York Times

[16] Kingma DP, Ba J (2015) Adam: a method for stochastic optimization. In: ICLR (Poster)

[17] Kumar A, Saumya S, Singh JP (2020) NITP-AI-NLP@ UrduFake-FIRE2020: multi-layer dense neural network for fake news detection in urdu news articles. In FIRE (Working Notes), pp 458–463

[18] Kumar A, Singh JP, Saumya S (2019) A comparative analysis of machine learning techniques for disaster-related tweet classification. In 2019 IEEE R10 Humanitarian Technology Conference (R10-HTC)(47129), IEEE, pp 222–227

[19] Kwon S, Cha M, Jung K, Chen W, Wang Y (2013) Prominent features of rumor propagation in online social media. In 2013 IEEE 13th International Conference on Data Mining, IEEE, pp 1103–1108

[20] Lewandowsky S, Ecker UK, Seifert CM, Schwarz N, Cook J (2012). Misinformation and its correction: continued influence and successful debiasing. Psychol Sci Public Interest.

[21] Liu H (2019) A location independent machine learning approach for early fake news detection. In 2019 IEEE International Conference on Big Data (Big Data), IEEE, pp 4740–4746

[22] Merity S, Keskar NS, Socher R (2018) Regularizing and optimizing LSTM language models. In: International conference on learning representations

[23] Merity S, Shirish Keskar N,Socher R (2018) An Analysis of Neural Language Modeling at Multiple Scales. arXiv e-prints

[24] Paka WS, Bansal R, Kaushik A, Sengupta S, Chakraborty T (2021). Cross-sean: a cross-stitch semi-supervised neural attention model for COVID-19 fake news detection. Appl Soft Comput.

[25] Patwa P, Sharma S, Pykl S, Guptha V, Kumari G, Akhtar S, Ekbal A, Das A, Chakraborty T (2021) Fighting an infodemic: COVID-19 fake news dataset. In: Chakraborty T, Shu K, Bernard HR, Liu H, Akhtar MS (eds) Combating online hostile posts in regional languages during emergency situation. CONSTRAINT 2021. Communications in Computer and Information Science, vol 1402. Springer, Cham, pp 21-29. 10.1007/978-3-030-73696-5_3

[26] Pennycook G, McPhetres J, Zhang Y, Lu JG, Rand DG (2020). Fighting COVID-19 misinformation on social media: experimental evidence for a scalable accuracy-nudge intervention. Psychol Sci.

[27] Reis JC, Correia A, Murai F, Veloso A, Benevenuto F (2019). Supervised learning for fake news detection. IEEE Intell Syst.

[28] Ruchansky N, Seo S, Liu Y (2017) Csi: a hybrid deep model for fake news detection. In: Proceedings of the 2017 ACM on Conference on Information and Knowledge Management, pp 797–806

[29] Sanh V, Debut L, Chaumond J, Wolf T (2019) Distilbert, a distilled version of bert: smaller, faster, cheaper and lighter. arXiv e-prints arXiv:1910.01108

[30] Saumya S, Singh JP (2018). Detection of spam reviews: a sentiment analysis approach. CSI Trans ICT.

[31] Saumya S, Singh JP (2020). Spam review detection using LSTM autoencoder: an unsupervised approach. Electron Commer Res.

[32] Shu K, Awadallah AH, Dumais S, Liu H (2020). Detecting fake news with weak social supervision. IEEE Intell Syst.

[33] Shu K, Mahudeswaran D, Wang S, Liu H (2020). Hierarchical propagation networks for fake news detection: investigation and exploitation. Proc Int AAAI Conf Web and Soc Media.

[34] Shu K, Sliva A, Wang S, Tang J, Liu H (2017). Fake news detection on social media: a data mining perspective. ACM SIGKDD Explor Newsl.

[35] Shu K, Wang S, Liu H (2019) Beyond news contents: The role of social context for fake news detection. In: Proceedings of the twelfth ACM International Conference on Web Search and Data Mining, pp 312–320

[36] Varol O, Ferrara E, Menczer F, Flammini A (2017). Early detection of promoted campaigns on social media. EPJ Data Sci.

[37] Vijjali R, Potluri P, Kumar S, Teki S (2020) Two stage transformer model for COVID-19 fake news detection and fact checking. In: Proceedings of the 3rd NLP4IF workshop on NLP for internet freedom: censorship, disinformation, and propaganda, pp 1–10

[38] Vosoughi S, Roy D, Aral S (2018). The spread of true and false news online. Science.

[39] Wang WY (2017) “Liar, Liar Pants on Fire”: a new benchmark dataset for fake news detection. In: Proceedings of the 55th annual meeting of the association for computational linguistics, vol 2: Short Papers, pp 422–426

[40] Wang Y, Yang W, Ma F, Xu J, Zhong B, Deng Q, Gao J (2020) Weak supervision for fake nws detection via reinforcement learning. Proc AAAI Conf Artif Intell 34:516–523

[41] Willmore A (2016) This analysis shows how viral fake election news stories outperformed real news on facebook

[42] Yang Z, Dai Z, Yang Y, Carbell J, Salakhutdinov RR, Le QV (2019). Xlnet: generalized autoregressive pretraining for language understanding. Advances in neural information processing systems.

[43] Zafarani R, Zhou X, Shu K, Liu H (2019) Fake news research: Theories, detection strategies, and open problems. In: Proceedings of the 25th ACM SIGKDD International Conference on Knowledge Discovery and Data Mining, pp 3207–3208

[44] Zhang X, Cao J, Li X, Sheng Q, Zhong L, Shu K (2021) Mining dual emotion for fake news detection

[45] Zellers R, Holtzman A, Rashkin H, Bisk Y, Farhadi A, Roesner F, Choi Y (2019) Defending against neural fake news. Adv Neural Inform Process Syst 32:1–12

[46] Zhou X, Zafarani R (2018) Fake news: a survey of research, detection methods, and opportunities. arXiv preprint arXiv:1812.00315

